# Immune Imbalance in Sickle Cell Anemia: Flow Cytometric Insights Into Regulatory T Cells and Neutrophil Dynamics

**DOI:** 10.1002/jcla.70227

**Published:** 2026-04-14

**Authors:** Rukiye Ölçüoğlu, Funda Tanrıkulu, Sevtap Kılınç, Eda Çakmak, Süheyl Asma, Can Boğa, Hakan Özdoğu, İlknur Pamuk

**Affiliations:** ^1^ Department of Physiology Faculty of Medicine, Başkent University Ankara Türkiye; ^2^ Division of Hematology, Department of Internal Medicine Faculty of Medicine, Çukurova University Adana Türkiye; ^3^ Department of Audiology Faculty of Health Sciences, Başkent University Ankara Türkiye; ^4^ Department of Hematology Dr. Turgut Noyan Application and Research Center, Başkent University Adana Türkiye

**Keywords:** flow cytometry, immune dysregulation, neutrophil activation, regulatory T cells (Tregs), sickle cell anemia

## Abstract

**Objective:**

To investigate immunophenotypic alterations in regulatory T cells (Tregs) and neutrophil dynamics in adult sickle cell anemia (SCA) patients during painful crises and steady state.

**Methods:**

Ninety‐three participants were included: 17 SCA patients in painful crisis, 27 in steady state, and 49 healthy controls. Flow cytometry was used to assess CD3^+^CD4^+^CD25^+^FoxP3^+^ Tregs and related subsets. Hematological parameters were evaluated by complete blood counts. Statistical analyses included group comparisons, multiple regression, and ROC curve analysis.

**Results:**

SCA patients exhibited significant reductions in CD25^+^ and CD4^+^ T cell subsets, despite preserved FoxP3^+^ Treg frequencies. White blood cell and neutrophil counts were elevated, especially during crisis. Neutrophil and lymphocyte percentages significantly predicted T cell subset levels. ROC analysis identified CD3^+^ and CD4^+^ percentages as strong classifiers of disease state.

**Conclusion:**

Despite numerical preservation of FoxP3^+^ Tregs, SCA is marked by impaired T cell activation and sustained innate immune activation. These immune shifts may relate to but do not directly determine end‐organ complications. Functional assays and longitudinal studies are needed to elucidate Treg competence, hydroxyurea effects, and age‐related immune changes in SCA.

## Graphical abstract

Flow cytometric analysis in sickle cell anemia reveals preserved Treg frequency despite decreased CD25 expression and increased neutrophil activity, highlighting a complex immune imbalance. The proportions of both neutrophil and lymphocyte cells have been shown to be a reliable predictor of CD3+ T cell levels, thus emphasizing the importance of innate‐adaptive immune crosstalk in the severity of disease.

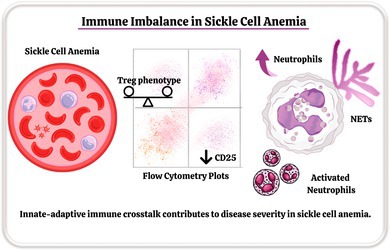

## 1 Introduction

Sickle‐cell anemia (SCA) is the most prevalent, severe monogenic disorder on a global scale. It is characterized by recurrent episodes of acute illness and progressive organ damage [1]. This condition arises from a mutation in the β‐globin gene, leading to the synthesis of abnormal hemoglobin S (HbS) [2]. Despite significant advancements in understanding the molecular and genetic mechanisms underlying SCA, therapeutic options remain limited, focusing primarily on symptom management rather than addressing the root causes of the disease.

Recent studies [3, 4, 5, 6, 7, 8] have emphasized the role of the immune system in the progression of SCA, which is increasingly recognized as an inflammatory condition associated with alterations in immune phenotype and function [7]. T regulatory cells (Tregs) have garnered attention for their anti‐inflammatory effects and capacity to modulate immune responses. Tregs have been identified as being pivotal in the maintenance of immune tolerance and the prevention of autoimmune diseases [9, 10]. In addition to the regulatory function of T cells, mounting evidence underscores the substantial contribution of innate immune cells, particularly neutrophils, to the persistent inflammation observed in SCA. Neutrophils in SCA exhibit a primed phenotype, with enhanced adhesiveness, oxidative activity, and the capacity to form neutrophil extracellular traps (NETs), especially during vaso‐occlusive crises (VOCs), contributing to endothelial injury and microvascular occlusion [3].

Despite the demonstrable involvement of the innate immune system in SCA [11] using both animal models and clinical studies, the role of the adaptive immune system in the pathophysiology of SCA remains poorly understood. Preliminary investigations have indicated alterations in the numbers, frequencies, and functions of T and B lymphocytes in SCA patients [6, 7, 8, 12]. However, further in‐depth and comprehensive research is required to elucidate the immune status of SCA patients and to determine the extent of dysfunction within specific immune cell subsets. The present study has therefore investigated the alterations in CD3, CD25, CD4, CD8, and CD4/CD8 ratio, as well as CD4^+^CD25^+^FoxP3^+^ cell populations. Furthermore, the study encompassed the analysis of leukocytes (neutrophils, lymphocytes, and monocytes), hemoglobin, and platelet counts. This comparative approach involved patients with SCA during painful crises and stable states, as well as healthy controls. The objective of this analysis was to elucidate their contributions to the pathophysiology of SCA.

## 2 Materials and Methods

A cross‐sectional study was prospectively conducted on patients diagnosed with SCA who were recruited from the regular attendees of the Hematology Clinic at the Dr. Turgut Noyan Application and Research Center, which is affiliated with Baskent University. Prior to participation, informed consent was obtained from the legal guardian of each patient and control. The study procedures were approved by the Başkent University Institutional Review Board for Non‐Interventional Clinical Studies (Approval No: 23/12, Date: 18/01/2023) and are in accordance with the Helsinki Declaration of 1975.

The study population comprised a total of 93 participants, including 17 patients in the painful crisis group, 27 patients in the steady‐state group, and 49 healthy volunteers. Patients with SCA were stratified into two groups based on their clinical status: Those experiencing an acute painful crisis and those in a steady state. Patients with SCA underwent a comprehensive review of their medical history and a detailed clinical examination, with a focus on specific complications associated with the disease. These included cerebrovascular events, avascular necrosis, retinopathy, hepatopathy, nephropathy, priapism, and pulmonary hypertension. Baseline demographic (age, sex) and clinical characteristics (history of vaso‐occlusive crises, hydroxyurea use, routine red cell exchange history, and selected complications such as cerebrovascular events, avascular necrosis, retinopathy, hepatopathy, nephropathy, priapism, pulmonary hypertension, alloimmunization) were systematically recorded and are presented in Table 1.

**TABLE 1:** Baseline demographic and clinical characteristics of the study participants.

Characteristic	HG (*n* = 49)	SCA_SS (*n* = 27)	SCA_PC (*n* = 17)
Age, years (mean ± SD)	—	40.31 ± 9.39	36.24 ± 9.46
Sex, *n* (%)			
Female	29 (59.2)	14 (51.9)	7 (41.2)
Male	20 (40.8)	13 (48.1)	10 (58.8)
History of VOC, *n* (%)	—		
No		5 (18.5)	2 (11.8)
1–2		18 (66.7)	9 (52.9)
≥ 3		4 (14.8)	6 (35.3)
Hydroxyurea use, *n* (%)	—		
No		8 (30.8)	4 (23.5)
Yes		18 (69.2)	13 (76.5)
Routine red cell exchange, *n* (%)	—		
No		25 (92.6)	10 (58.8)
Yes		2 (7.4)	7 (41.2)
Alloimmunization, *n* (%)	—		
No		20 (74.1)	15 (88.2)
Yes		7 (25.9)	2 (11.8)
Stroke (SVO), *n* (%)	—		
No		26 (96.3)	14 (82.4)
Yes		1 (3.7)	3 (17.6)
Avascular necrosis, *n* (%)	—		
No		14 (51.9)	6 (35.3)
Yes		13 (48.1)	11 (64.7)
Retinopathy, *n* (%)	—		
No		23 (88.5)	17 (100)
Yes		3 (11.5)	—
Hepatopathy, *n* (%)	—		
No		22 (81.5)	11 (68.8)
Yes		5 (18.5)	5 (31.2)
Nephropathy, *n* (%)	—		
No		16 (59.3)	12 (75)
Yes		11 (40.7)	4 (25)
Priapism, *n* (%)	—		
No		26 (96.3)	13 (81.3)
Yes		1 (3.7)	3 (18.7)
Pulmonary hypertension, *n* (%)	—		
No		22 (81.5)	14 (87.5)
Yes		5 (18.5)	2 (12.5)

*Note:* The data are presented as the mean ± standard deviation (SD) for age and as the number (n) with the percentage (%) for categorical variables. The healthy control group was not assessed for disease‐specific clinical characteristics (indicated by “‐”).

*Abbreviations:* HG, healthy group; SCA_PC, sickle cell anaemia in painful crises; SCA_SS, sickle cell anaemia in a steady state; SVO, cerebrovascular occlusion; VOC, vaso‐occlusive crisis.

The inclusion criteria for participation in the study were as follows: Individuals must have been 18 years of age or older, have a confirmed diagnosis of SCA, and provide informed consent to participate. Participants were excluded from the study if they were pregnant, had a confirmed diagnosis of cancer or autoimmune disease, presented with signs of acute infection, had undergone stem cell transplantation, were receiving treatment for SCA with investigational drugs such as crizanlizumab, inclacumab, or etavopivat as part of a clinical trial, or had infections, chronic inflammatory conditions other than SCA, renal or cardiac disease, rheumatoid arthritis, hypothyroidism, diabetes mellitus, or were undergoing steroid therapy.

### 2.1 Sample Collection and Laboratory Assessments

The collection of samples was undertaken with the objective of conducting a complete blood count (CBC) and a flow cytometric analysis for subgrouping of T lymphocytes. The samples were collected using potassium ethylene diamine tetra‐acetic acid (K2‐EDTA, 1.2 mg/mL) as an anticoagulant. Laboratory assessments were then conducted immediately. The laboratory investigations included a CBC performed using the CELL‐DYNE Rubby analyser (Wiesbaden, Germany).

### 2.2 Flow Cytometric Evaluation of CD4 ^+^ CD25 ^+^ T Lymphocytes and Lymphocyte Subset

CD4^+^CD25^+^ T lymphocytes were assessed using flow cytometry. For each analysis, 100 μL of peripheral blood was mixed with 10 μL of the respective fluorochrome‐labeled antibodies, with appropriate isotype controls included. Samples were incubated at room temperature for 20 min in the dark. A customized single‐tube panel consisting of CD45KRO, CD3PB, CD4FITC, CD8PC7, CD25A700, and FoxP3 APC was designed specifically for this study. All reagents and consumables were obtained from Beckman Coulter (Marseille, France).

Data acquisition was performed on a Navios 3L10C flow cytometer (San Jose, USA), equipped with three lasers and capable of 10‐color analysis. A total of a minimum of 100,000 events was acquired per tube, and data were analyzed using Kaluza software.

Firstly, unlysed erythrocytes were excluded prior to analysis. During the analysis, lymphocytes were initially gated on the CD45/SS plot, followed by the selection of CD3‐positive T cells within the lymphocyte population. Within this CD3^+^ population, cells that were brightly positive for CD4 and CD25 were identified, and subsequently, FoxP3‐positive cells were gated. Additionally, the CD4/CD8 ratio was calculated (Figure S1). The results were reported as both percentages and absolute counts per microliter.

### 2.3 Statistical Analysis

The sample size of the study was determined using the GPower3.1 program. The effect size was determined as Cohen's f = 0.40, the error level as α = 0.05, and the test power as (1‐β) = 0.90, and the total sample size required was determined as at least 84 participants.

Statistical analyses were conducted by IBM SPSS Statistics Version 25.0. Armonk. NY: IBM Corp. To evaluate the normality of distribution Kolmogorov–Smirnov test, and for homogeneity of variances Levene test were used. Independent sample *t*‐test, Mann–Whitney U test, and Kruskal–Wallis test were used in comparisons between groups. Multiple linear regression analysis was applied to examine the parameters affecting flow variables. The receiver operating characteristic (ROC) curve analysis was used to calculate sensitivity, specificity, the area under the curve (AUC), and the optimal cut‐off value of parameters. The level of statistical significance was considered *p* < 0.05 in all data.

## 3 Results

The results are presented by means of a summary of the findings from both flow cytometry and hemogram analyses, with descriptive statistics provided in Tables 2 and S1, respectively. These tables present the mean ± standard deviation (SD) and median (interquartile range, IQR) for the measured parameters in three distinct groups: Patients in a pain crisis (SCA‐PC), steady state patients (SCA‐SS), and a healthy control group (HG).

**TABLE 2:** Flow cytometric analysis of lymphocyte subsets and regulatory t cells in healthy controls and patients with sickle cell anemia.

Parameters	HG (*n* = 49)		SCA_PC (*n* = 17)		SCA_PC – HG		SCA_SS (*n* = 27)		SCA_SS—HG		SCA (*n* = 44)		SCA‐HG	
	Mean ± SD	Median (IQR)	Mean ± SD	Median (IQR)	Test statistics	*p*	Mean ± SD	Median (IQR)	Test statistics	*p*	Mean ± SD	Median (IQR)	Test statistics	*p*
Lymphocyte %	33.27 ± 9.04	32.6 (14.12)	38.32 ± 10.61	37.23 (14.81)	1.897^t^	0.062	37.21 ± 11.59	39.47 (17.14)	1.643^t^	0.105	37.64 ± 11.11	39.09 (16.24)	2.089^t^	**0.039** *
CD3+ %	75.07 ± 7.37	76.02 (8.39)	57.49 ± 13.02	57.31 (21.16)	‐6.851^t^	**< 0.001** ***	63.60 ± 9.48	63.2 (16.32)	‐5.857^t^	**< 0.001** ***	61.24 ± 11.25	60.84 (17.97)	‐6.932^t^	**< 0.001** ***
CD3+ CD4+ CD25+ %	3.38 ± 1.74	3.03 (1.75)	2.56 ± 1.95	1.64 (2.92)	‐1.936^Z^	**0.053** ^**a**^	2.20 ± 1.17	2.16 (1.6)	−3.077^Z^	**0.002** **	2.34 ± 1.51	2.10 (1.98)	−3.197^Z^	**0.001** ***
CD3+ CD4+ CD25+ count	861.12 ± 393.53	793 (528)	521.12 ± 372.09	381 (588)	−3.111^t^	**0.003** **	511.15 ± 302.65	481 (411)	−4.009^t^	**< 0.001** ***	515.00 ± 326.99	460 (465)	−4.321^Z^	**< 0.001** ***
CD3+ CD4+ CD25+ FoxP3+ %	4.91 ± 2.24	4.96 (2.71)	4.72 ± 1.95	4.76 (3.13)	−0.305^t^	0.762	4.41 ± 1.77	4.66 (2.97)	−0.993^t^	0.324	4.53 ± 1.83	4.67 (2.94)	−0.883^t^	0.379
CD3+ CD4+ CD25+ FoxP3+ count	1230.61 ± 529.42	1092 (857)	960.53 ± 373.58	860 (504)	−1.606^Z^	0.108	1030.15 ± 484.21	1023 (819)	−1.416^Z^	0.157	1003.25 ± 441.45	925 (683)	−1.847^Z^	0.065
CD3+ CD4+ (T helper) %	45.67 ± 8.42	45.43 (7.95)	35.21 ± 9.96	33.89 (12.38)	−4.208^t^	**< 0.001** ***	38.97 ± 7.12	38.64 (9.71)	−3.499^t^	**0.001** ***	37.52 ± 8.43	37.28 (10)	−4.372^Z^	**< 0.001** ***
CD3+ CD8+ (Cytotoxic T) %	25.96 ± 6.97	26.31 (9.98)	17.70 ± 5.93	17.67 (8.23)	−4.359^t^	**< 0.001** ***	20.53 ± 7.47	19.46 (14.08)	−3.164^t^	**0.002** **	19.44 ± 6.99	18.89 (8.98)	−4.497	**< 0.001** ***
CD4/CD8 ratio	1.93 ± 0.80	1.71 (0.92)	2.36 ± 1.63	1.90 (1.2)	−0.784^Z^	0.433	2.19 ± 0.88	2.16 (1.67)	−1.232^Z^	0.218	2.25 ± 1.21	2.07 (1.45)	−1.285^Z^	0.199
Treg (CD4+ CD25+ FoxP3+) % in total cells	1.18 ± 0.58	1.03 (0.79)	0.97 ± 0.43	0.89 (0.44)	−1.063^Z^	0.288	0.99 ± 0.46	1.01 (0.81)	−1.091^Z^	0.275	0.99 ± 0.45	0.95 (0.56)	−1.331^Z^	0.183
CD3+ CD4+ CD25+ % in total cells	0.81 ± 0.41	0.74 (0.52)	0.50 ± 0.33	0.42 (0.55)	−2.791^t^	**0.007** **	0.50 ± 0.29	0.48 (0.38)	−3.528^t^	**0.001** ***	0.50 ± 0.30	0.45 (0.45)	−3.955^Z^	**< 0.001** ***

*Note:* The data are presented as mean ± standard deviation and median (interquartile range‐IQR). Group comparisons were performed using either the Independent Samples *t*‐test (marked ‘t’) or the Mann–Whitney U test (marked ‘Z'), as appropriate.

*Abbreviations:* HG, healthy controls; SCA, all SCA patients combined; SCA_PC, SCA patients in painful crisis; SCA_SS, SCA patients in steady state.

* *p* < 0.05.

** *p* < 0.01.

*** *p* < 0.001.

^a^ Marginally significant.

### 3.1 Flow Cytometric Analysis and CBC Results

**FIGURE 1:**
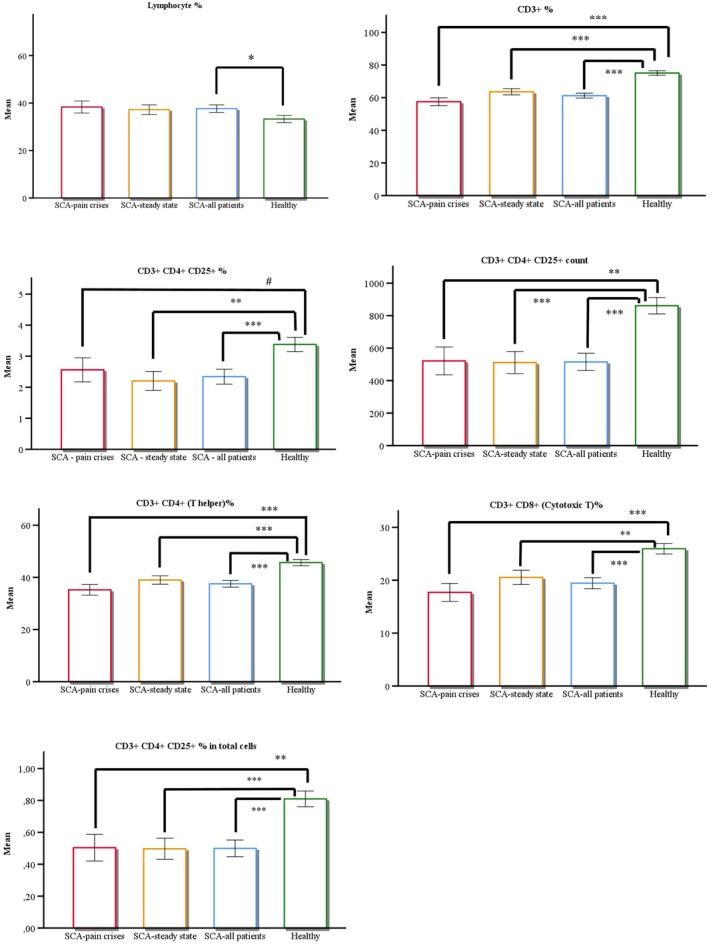
Significant alterations in T lymphocyte subsets and regulatory T cells in sickle cell anemia patients during painful crisis compared to steady state and healthy controls. The bar graphs demonstrate significant differences in (A) Lymphocyte %, (B) CD3+ %, (C) CD3 + CD4 + CD25+ %, (D) CD3 + CD4 + CD25+ cell count, (E) CD3 + CD4+ T helper cells, (F) CD3 + CD8+ cytotoxic T cells, and (G) CD3 + CD4 + CD25+ percentage in total cells, among healthy control (HG, n = 49), steady‐state SCA (SCA‐SS, n = 27), and painful crisis SCA (SCA‐PC, n = 17) groups. The data are presented as the mean with error bars representing the standard deviation (SD). Data represent mean ± SD. Statistical comparisons were performed using the Independent Samples t‐test or the Mann–Whitney U test. *p < 0.05, **p < 0.01, ***p < 0.001 indicate significant differences vs. *healthy controls. # denotes marginal significance (p = 0.053)*.

The analysis of flow cytometry data demonstrated that, compared to the healthy control group (HG), patients in a pain crisis (SCA‐PC) had significantly altered immunophenotypic profiles. Specifically, SCA‐PC patients exhibited a significantly lower percentage of CD3^+^ cells (*p* < 0.001), a reduced CD25 count (*p* = 0.003), a decreased percentage of CD3^+^CD4^+^ cells (*p* < 0.001), an altered CD4/CD8 ratio (*p* < 0.001), and a diminished percentage of CD25 in total cells (*p* = 0.007). Similarly, steady state patients (SCA‐SS) also exhibited significant differences from HG, with notably lower levels of CD3^+^% (*p* < 0.001), CD25% (*p* = 0.002), CD25 count (*p* < 0.001), and CD3^+^CD4^+^% (*p* < 0.001), as well as an altered CD4/CD8 ratio (*p* = 0.002) and reduced CD25% in total cells (*p* = 0.001). No significant differences were observed in CD3^+^CD4^+^CD25^+^FoxP3^+^ Treg frequencies across the groups (all *p* > 0.05), and CD8^+^ cytotoxic T‐cell percentages remained comparable among all cohorts (*p* > 0.05). These findings are detailed in Table 2 and graphically illustrated in Figure 1. Hematological parameters (see Table S1) confirmed the typical SCA profile, including elevated white blood cell (WBC) counts in SCA‐PC (17.75 ± 10.20 vs. HG 7.25 ± 1.76, *p* < 0.001) and markedly reduced hemoglobin levels in both SCA groups (*p* < 0.001).

### 3.2 Subgroup Analysis by Complications

Subgroup analyses based on clinical complications (Tables S2 and S3) were conducted to assess potential associations between complications and immune parameters. These parameters have been stratified by the presence or absence of various complications (e.g., avascular events, retinopathy, etc.). The results of the flow cytometry analysis (Table S2) indicate that the majority of the markers did not demonstrate statistically significant differences between patients with and without complications. However, exceptions to this were observed in the cases of hepatopathy (reduced CD3^+^ %, *p* = 0.041) and priapism (reduced CD3^+^ %, *p* = 0.046), suggesting that, while there was limited variation, it was nonetheless noteworthy. Furthermore, the hemogram analysis (Table S3) revealed variable patterns. For instance, hepatopathy was associated with significant changes in WBC (*p* = 0.008), neutrophil (*p* = 0.001), lymphocyte (*p* = 0.004), and HCT values (*p* = 0.015). Furthermore, both the avascular and retinopathy subgroups exhibited significant or borderline significant variations in monocyte percentages (*p* = 0.044 and *p* = 0.050, respectively). The routine red cell exchange subgroup also exhibited significant differences in WBC (*p* = 0.033) and monocyte percentage (*p* = 0.034). Conversely, other complications had no significant impact.

### 3.3 Regression Analyses

Multiple linear regression analyses were conducted to ascertain the variables that significantly predict various immunophenotypic parameters (Table 3). For CD3%, both lymphocyte percentage (β = 0.525, *p* = 0.001) and neutrophil percentage (β = 0.615, *p* < 0.001) were significant positive predictors, indicating that higher lymphocyte and neutrophil proportions were associated with increased total T‐cell percentages. Conversely, the regression model for CD25% exhibited an inverse correlation with lymphocyte percentage (β = −0.432, *p* = 0.004), indicating that increased lymphocyte levels are associated with diminished CD25 expression among total cells. For CD4%, both lymphocyte (β = 0.418, *p* = 0.002) and neutrophil percentages (β = 0.309, *p* = 0.006) were significant predictors, whereas CD8% was predicted solely by neutrophil percentage (β = 0.512, *p* = 0.001). A similar trend was observed in the prediction of total cell FoxP3%, where lymphocyte percentage emerged as the sole significant predictor (β = 0.391, *p* = 0.009). Furthermore, both lymphocyte and neutrophil percentages were found to be significant predictors of CD25% within total cells, with regression coefficients of 0.442 and 0.327, respectively, and both *p* < 0.05. Collectively, these results demonstrate a functional association between innate (neutrophil) and adaptive (lymphocyte) immune components, indicating that the balance of these cell populations influences T‐cell subset variability in SCA.

**TABLE 3:** Multiple linear regression analysis for predictors of t cell subset percentages.

CD3+ %	*b*	S(b)	BETA	VIF	*t*	*p*
Constant	19.229	9.940				
Lymphocyte ratio %	0.525	0.152	0.462	2.300	3.444	0.001
Neutrophil %	0.615	0.104	0.797	2.300	5.940	< 0.001
$R^{2}$ = 0.295 *(F* = 18.840 *p* < 0.001) *d* = 1.689						
**CD3+ CD4+ CD25+ %**	***b***	***S(b)***	***BETA***	***VIF***	***t***	***p***
Constant	4.578	0.615				
Lymphocyte ratio %	−0.048	0.017	−0.288	1.000	−2.864	0.005
$R^{2}$ = 0.083 *(F* = 8.202 *p* = 0.005) *d* = 1.353						
**CD3+ CD4+ (T helper)%**	***b***	***S(b)***	***BETA***	***VIF***	***t***	***p***
Constant	10.446	8.676				
Lymphocyte ratio %	0.361	0.133	0.397	2.300	2.713	0.008
Neutrophil %	0.372	0.090	0.603	2.300	4.119	< 0.001
$R^{2}$ = 0.161 *(F* = 8.561 *p* < 0.001) *d* = 1.858						
**CD3+ CD8+ (Cytotoxic T)%**	***b***	***S(b)***	***BETA***	***VIF***	***t***	***p***
Constant	3.186	7.324				
Neutrophil %	0.252	0.076	0.496	2.300	3.303	0.001
$R^{2}$ = 0.118 *(F* = 6.012 *p* = 0.004) *d* = 1.950						
**Treg (CD4+ CD25+ FoxP3+) % in total cells**	***b***	***S(b)***	***BETA***	***VIF***	***t***	***p***
Constant	0.556	0.189				
Lymphocyte ratio %	0.015	0.005	0.294	1.000	2.931	0.004
$R^{2}$ = 0.086 *(F* = 8.589 *p* = 0.004) *d* = 1.809						
**CD3+ CD4+ CD25+ % in total cells**	***b***	***S(b)***	***BETA***	***VIF***	***t***	***p***
Constant	−0.467	0.380				
Lymphocyte ratio %	0.017	0.006	0.438	2.300	2.870	0.005
Neutrophil %	0.017	0.004	0.416	2.300	2.727	0.008
$R^{2}$ = 0.091 *(F* = 4.491 *p* = 0.014) *d* = 1.350						

*Note:* Multiple linear regression models were constructed, with the indicated T cell parameter designated as the dependent variable. In the models, the lymphocyte percentage and neutrophil percentage were included as independent variables. The following were included in the models: b, unstandardized coefficient; S(b), standard error of the coefficient; BETA, standardized coefficient; VIF, variance inflation factor (for multicollinearity assessment); R^2^, coefficient of determination; and d, Durbin‐Watson statistic (for autocorrelation assessment).

### 3.4 ROC Curve Analysis

To assess the discriminatory power of the studied parameters, ROC curve analyses were conducted. In the flow cytometry analysis, parameters such as CD3%, CD3CD4%, and the CD3^+^CD4^+^CD25^+^ count demonstrated high AUC values (e.g., CD3% AUC = 0.849), indicating their robust ability to distinguish between patient groups. The ROC analysis yielded optimal cut‐off values for each parameter, accompanied by the relevant sensitivity and specificity figures (Table 4). Furthermore, the hemogram parameters, encompassing WBC count, neutrophil percentage, lymphocyte percentage, HGB, HCT, and PLT, also exhibited significant diagnostic utility (AUC range: 0.75–0.90), as evidenced by their respective ROC curve findings.

**TABLE 4:** ROC curve analysis of flow cytometric and hemogram parameters for discriminating SCA patients from healthy controls.

Parameters (flow)	AUC	SE	95% CI	Cut‐off value	Sensitivity (%)	Specificity (%)	*p*
Lymphocyte %	0.629	0.059	0.52–0.73	35.58	61.36	65.31	**0.029**
CD3+ %	0.849	0.041	0.76–0.92	71.69	86.36	77.57	**< 0.001**
CD3+ CD4+ CD25+ %	0.693	0.056	0.59–0.78	2.57	70.45	69.39	**0.001**
CD3+ CD4+ CD25+ count	0.760	0.050	0.66–0.84	578	68.18	77.55	**< 0.001**
CD3+ CD4+ (T helper)%	0.785	0.049	0.69–0.86	40.28	72.73	83.67	**< 0.001**
CD3+ CD8+ (Cytotoxic T)%	0.747	0.051	0.65–0.83	23.87	77.27	65.31	**< 0.001**
CD3+ CD4+ CD25+ % in total cells	0.738	0.052	0.64–0.82	0.53	65.91	75.51	**< 0.001**
**Parameters (Hemogram)**	**AUC**	**SE**	**95% CI**	**Cut‐off value**	**Sensitivity (%)**	**Specificity (%)**	***p***
WBC (White blood cells)	0.827	0.049	0.74–0.90	9.82	70.45	89.80	**< 0.001**
Neutrophil	0.800	0.049	0.70–0.88	43.20	54.55	100	**< 0.001**
Lymphocyte	0.766	0.052	0.67–0.85	41.40	61.36	89.80	**< 0.001**
HGB (Hemoglobin)	0.953	0.027	0.89–0.99	12.20	95.45	91.84	**< 0.001**
HCT (Hematocrit)	0.896	0.039	0.82–0.95	35.10	86.36	91.84	**< 0.001**
PLT (Platelet)	0.707	0.059	0.60–0.80	336	54.55	93.88	**0.001**

*Note:* The analysis evaluates the ability of each parameter to distinguish all SCA patients (combined crisis and steady state groups) from healthy controls. *p* < 0.05.

*Abbreviations:* AUC, area under the ROC curve; CI, confidence interval; SE, standard error.

## 4 Discussion

In the present study, we have explored the immunopathological role of Treg cells in SCA. To this end, rigorous flow cytometric and hematological assessments were conducted across distinct clinical states, namely painful crisis SCA, steady state SCA, and healthy controls. Utilizing this established knowledge, the present study sought to elucidate the specific role and alterations of Treg cells in SCA.

FoxP3, the master transcription factor of Treg cells, plays a fundamental role in maintaining immune tolerance by regulating gene programs that define and sustain Treg identity and function [13, 14]. FoxP3 predominantly functions as a transcriptional repressor, influencing Treg functionality by regulating an extensive enhancer network established during their development [15, 16]. However, its function is not independent, as FoxP3 interacts with multiple cofactors, which are not only required for Treg differentiation but also directly regulated by FoxP3 itself [17, 18, 19]. This reciprocal regulation enables Tregs to maintain immune homeostasis while adapting to inflammatory conditions. FoxP3 stability is imperative for the functionality of Tregs, and its downregulation has been linked to autoimmunity and chronic inflammation [20, 21, 22, 23, 24, 25]. Nevertheless, Tregs must retain some degree of plasticity to effectively control immune responses. For instance, Treg expression of the transcription factor T‐bet is necessary for regulating Th1‐driven inflammation while maintaining FoxP3 expression [26]. In view of the fact that SCA is characterized by persistent inflammation and immune activation, it remains unclear whether Treg plasticity is beneficial or detrimental in this context. In consideration of the pivotal functions of FoxP3 in immune tolerance, its stability or dysregulation assumes particular relevance in chronic inflammatory conditions such as SCA.

The present study demonstrated that (Table 2), while the percentage of CD3^+^CD4^+^CD25^+^FoxP3^+^ Tregs did not differ significantly between SCA patients and healthy controls (*p* > 0.05), the percentage of CD3^+^CD4^+^CD25^+^ cells was significantly reduced (*p* < 0.01). This finding suggests that FoxP3^+^ Tregs may maintain numerical stability, but early differentiation or IL‐2‐dependent expansion may be impaired, given the essential role of CD25 (IL‐2Rα) in Treg survival [27]. CD25 constitutes the α‐chain of the interleukin‐2 receptor and plays a pivotal role in IL‐2‐mediated signaling required for the survival, proliferation, and suppressive function of regulatory T cells. Reduced CD25 expression may therefore indicate diminished IL‐2 responsiveness within the T‐cell compartment, even when FoxP3+ Treg frequencies appear numerically preserved. As demonstrated in previous studies, IL‐2 signaling is critical for maintaining Treg homeostasis and functional competence. Impaired IL‐2 receptor signaling may lead to a state in which Tregs are present but functionally insufficient to adequately control inflammatory responses [28, 29]. Within the framework of SCA, a persistent inflammatory milieu has been demonstrated to perpetuate FoxP3 expression, concurrently modulating IL‐2‐dependent regulatory pathways. Furthermore, the observed decrease in CD3^+^CD4^+^CD25^+^ cells (*p* < 0.01) and the reduced CD3^+^CD4^+^ T helper cell percentage (*p* < 0.001) are consistent with studies reporting alterations in adaptive immunity in SCA patients [30, 31]. This finding is of particular significance given the critical role of IL‐2 signaling in the survival and expansion of Tregs. Disruptions in CD25 expression may signify an impaired capacity of SCA patients to maintain a fully functional Treg compartment. Given that CD25 is a key marker for both Treg activation and effector T cell proliferation, its reduction could reflect an overall dysregulation in T cell homeostasis. Moreover, while the present study lends support to the notion that FoxP3 expression is maintained, prior studies have reported Treg activation marker upregulation (CTLA‐4, CD39) in SCA patients, suggesting that Tregs may undergo compensatory activation in response to chronic inflammation [6, 7]. Despite these phenotypic shifts, Treg suppressive function was found to be comparable to that of healthy controls, raising the possibility that SCA‐associated inflammation modifies Treg phenotype without fully compromising function [6].

When considered as a whole, these data emphasize that neutrophils in SCA are not merely bystanders but rather central contributors to the immunopathogenesis of the disease. The hypothesis that their persistent activation may contribute not only to acute VOC episodes but also to chronic, low‐grade inflammation is one that merits further investigation. This, in turn, may have a potential effect on Treg plasticity, cytokine balance, and overall immune homeostasis in SCA. However, it should be noted that the current data set does not allow for conclusions to be drawn regarding the occurrence of complications in the end organs. This is an issue that necessitates dedicated clinical studies.

The adaptive immune system in SCA displays notable alterations beyond Treg cells. Some studies have reported increased total T lymphocytes and CD8^+^ T cells in steady‐state SCA patients, whereas others have found reductions in total T cells, including CD4^+^ and CD8^+^ subsets [30, 31, 32]. The present study concurs with the proposition of CD4^+^ T cell depletion in SCA, particularly during vaso‐occlusive crises (VOC) [32], though no significant difference in the CD4/CD8 ratio was observed (*p* > 0.05), which is in contrast to the reports of a lower ratio in VOC patients [32]. It is noteworthy that an increase in central memory CD4^+^ T cells has been observed in SCA patients, which may indicate a shift in T cell memory compartmentalization [33]. Furthermore, studies have identified elevated levels of leukocytes and neutrophils in both steady‐state SCA and VOC patients [32], thereby substantiating the notion that SCA is predominantly driven by chronic immune activation rather than a conventional immunodeficiency [32].

In addition to the adaptive immune alterations, mounting evidence indicates that innate immunity, particularly that of the neutrophils, plays a significant role in the pathogenesis of SCA. In accordance with this, the present study, in conjunction with extant literature, emphasizes the pivotal role of neutrophils as active effectors in the pathogenesis of SCA. Neutrophils are increasingly recognized as central mediators of vascular inflammation and tissue injury in SCA, acting through mechanisms such as adhesive molecule upregulation, oxidative burst, and neutrophil extracellular trap (NET) formation [3, 34]. This proinflammatory neutrophil activity in question has been shown to correlate with endothelial dysfunction, VOC frequency, and overall disease severity. Musa et al. (2022) [32] reported significantly elevated neutrophil percentages even in steady‐state SCA patients, attributing this partly to chronic subclinical inflammation and IL‐8–mediated chemotaxis. Their findings further demonstrated that IL‐2 levels were elevated in both steady‐state and VOC conditions, without parallel increases in CD4^+^ T cells, suggesting the presence of dysregulated IL‐2 signaling or altered T cell receptor responsiveness. These results align with our observation that neutrophil and lymphocyte percentages are predictive of increased CD3^+^ T cell percentages, indicating that innate immune activation may influence adaptive T cell dynamics. Moreover, Marchesani et al. (2023) [35] demonstrated that neutrophils in SCA exhibit an activated phenotype even in basal conditions, with enhanced adhesiveness that worsens during VOC. Elevated absolute neutrophil counts have been repeatedly linked to more severe clinical outcomes, as previously reported by Anyaegbu et al. (1998) [36] and Fadlon et al. (1998) [37]. The neutrophil response is further intensified by hemolysis‐related DAMPs (e.g., free heme, hemoglobin), which stimulate innate immune pathways such as TLR4 and inflammasome signaling [34]. This self‐amplifying loop perpetuates inflammation and primes the immune system even in the absence of overt infection. When considered as a whole, these data emphasize that neutrophils in SCA are not merely bystanders but rather central contributors to the immunopathogenesis of the disease. It is hypothesized that their persistent activation may contribute not only to acute VOC episodes but also to chronic, low‐grade inflammation. This, in turn, may have a potential effect on Treg plasticity, cytokine balance, and overall immune homeostasis in SCA.

The present study also examined the relationships between flow cytometric parameters and clinical variables through regression analysis. For instance, the multiple linear regression model revealed that increases in lymphocyte percentage and neutrophil percentage were significantly associated with higher CD3^+^ T cell percentages (β = 0.525, *p* = 0.001; and β = 0.615, *p* < 0.001, respectively). These findings emphasize the contribution of both adaptive and innate immune components in SCA pathogenesis. The correlation between neutrophil percentages and T‐cell parameters observed in the regression analysis may reflect more than a simple statistical association. One potential explanation for this phenomenon is a relative reduction in lymphocyte proportions within the leukocyte differential count, resulting from marked neutrophilia during painful crises. However, it is noteworthy that neutrophils themselves may also exert regulatory effects on adaptive immune responses. Activated neutrophils have been shown to generate reactive oxygen species and proteolytic mediators capable of modulating T‐cell proliferation and survival. Such mechanisms have been described in inflammatory conditions in which neutrophils influence T‐cell viability and activation, thereby contributing to immune dysregulation [38, 39]. It is important to note that these mechanisms are not mutually exclusive and may collectively contribute to the altered immune balance observed in SCA. In the context of SCA, it has been demonstrated that chronic inflammation and recurrent vaso‐occlusive episodes trigger sustained activation of innate immune cells, such as neutrophils [40, 41, 42]. These cells not only contribute to endothelial injury but also appear to correlate with alterations in T cell populations. Elevated white blood cell (WBC) and neutrophil counts, commonly observed during painful crises, coupled with reductions in hemoglobin (HGB) and hematocrit (HCT) values (Table S1), further support the hypothesis that SCA is driven by persistent immune activation rather than classic immunodeficiency.

In view of the marked heterogeneity of the clinical manifestations of SCA, subgroup analyses were conducted on the basis of complications to facilitate more detailed exploration of immune alterations. The subgroup analysis (Table S2 and Table S3) demonstrates that the distribution of immune cells is subject to variation in the presence of complications. For example, in patients with avascular complications or retinopathy, certain flow parameters—such as CD3^+^ and CD25^+^ expression—tend to show further changes, although not all differences reach statistical significance. This trend is consistent with recent reports linking elevated inflammatory markers and altered immune profiles with specific SCA complications (e.g., increased IL – 10 production in patients with leg ulcers) [32]. In accordance with these findings, it was also observed that the levels of Tregs did not differ significantly between patients with and without nephropathy or hepatopathy. This finding indicates that the numerical stability of Tregs alone may not be a reliable predictor of end‐organ damage in SCA. It is evident that organ complications, such as nephropathy and hepatopathy, are the result of multifactorial mechanisms. These include repeated vaso‐occlusive events, oxidative stress, and endothelial dysfunction. Rather than being directly attributable to Treg alterations alone, these complications are the result of a combination of factors [1, 43]. Furthermore, it has been hypothesized that Treg dysfunction may occur independently of alterations in cell frequency, as certain studies have reported functional or phenotypic alterations in Tregs, including increased CTLA‐4 and CD39 expression, along with reduced expression of HLA‐DR and CCR7, indicating functional or phenotypic alterations without significant changes in absolute numbers [6]. The present findings lend support to the hypothesis that quantitative assessments of Tregs may be inadequate for reflecting their immunomodulatory role in SCA‐related complications. The performance of functional assays and phenotypic profiling of Treg subsets could provide greater insight into their potential involvement in chronic organ injury.

In addition, ROC curve analyses (Table 4) show that several flow cytometric parameters have high discriminatory power. In particular, CD3^+^ T cell percentage (AUC = 0.849) and CD3^+^CD4^+^ T helper cell percentage (AUC = 0.785) emerged as strong candidates for discriminating between disease states. These findings are consistent with those reported by Patel et al. [44], who reported an AUC of 0.851 based on naïve and memory T cell profiles to predict crisis status in SCA. In addition, in a manner analogous to our regression analysis linking innate (neutrophils) and adaptive (T cells) parameters, Patel et al. (2023) [44] also employed a logistic regression model to derive a composite immunophenotypic score for SCA complications. Despite the differences in the marker panels, both studies underscore the efficacy of multivariate immune profiling in elucidating disease dynamics and potentially informing patient monitoring.

The present findings contribute to the evolving understanding of immune dysregulation in SCA. While FoxP3+ Tregs appear to be numerically preserved, their functional capacity, subset distribution, and migratory abilities may be altered in response to chronic inflammation. The observed discrepancy between our findings and those of previous studies reporting increased Tregs in SCA suggests that Treg adaptation might be dependent on disease severity, patient heterogeneity, or methodological differences in immunophenotyping. It is also noteworthy that our study employed a single‐tube panel, which provided a highly standardized approach for Treg identification. The functional implications of these findings necessitate further investigation. Despite the preservation of FoxP3+ Treg percentages, there is a possibility that Treg function might be compromised in SCA. Prior studies suggest that, while Tregs in SCA exhibit increased activation, their suppressive capacity remains unchanged compared to controls [6]. However, it remains unclear whether this observation holds true across different clinical manifestations of the disease.

The present study is subject to several limitations that must be given due consideration. Firstly, the overall sample size was limited, and subgroup analyses based on specific complications (e.g., hepatopathy, nephropathy) were underpowered, potentially obscuring subtle differences. Furthermore, the exclusion of genotype and hemoglobin electrophoresis data from the analysis renders the phenotypic characterization of the patients based solely on clinical criteria, as opposed to molecular classification. Consequently, it is not possible to attribute immune alterations directly to specific SCA genotypes. As the absence of the hemoglobin genotype from the dataset precluded the establishment of a correlation between phenotype and genotype, this issue could be addressed in future multicenter studies. The study groups were heterogeneous, with some patients receiving hydroxyurea therapy, which might have influenced immune profiles. It is also important to consider the potential influence of hydroxyurea treatment on T cell dynamics and Treg functional profiles, as suggested in previous studies [45, 46]. It is recommended that future studies employ a stratified approach, categorizing participants into separate hydroxyurea‐treated and untreated groups, with the objective of elucidating the observed effects. Moreover, it is possible that age‐related variations in lymphocyte populations, particularly CD4^+^ and CD8^+^ subsets, may have influenced the results obtained, given the established knowledge that lymphocyte dynamics undergo changes with age [47]. Although the analysis did not delve into the intricacies of hydroxyurea exposure, prior studies [45] indicated that HU therapy may modify T‐cell distribution, particularly central memory subsets. This observation necessitates further investigation in larger cohorts. Furthermore, our analysis focused exclusively on Treg cells without evaluating other key adaptive immune components such as Th17 cells. While the phenotypic analysis provides valuable insight, the use of functional assays such as suppression capacity or cytokine profiling (e.g., IL‐10, TGF‐β production) would be instrumental in evaluating the true regulatory competence of Tregs in this context. Finally, the assessment of Tregs was primarily numerical and percentage‐based, with no investigation of their functional capacities or the diversity among different Treg subsets, which could provide further insights into their role in SCA.

In consideration of the established correlation between FoxP3 downregulation and Treg plasticity, on the one hand, and autoimmune conditions and chronic inflammation, on the other [20, 21, 22, 23, 24, 25], it can be hypothesized that SCA‐associated immune activation may impact Treg function, albeit without necessarily affecting FoxP3 expression. Moreover, earlier studies have demonstrated that Tregs require a balance between stability and adaptability to effectively regulate immune responses, particularly in Th1‐driven inflammation, where T‐bet expression in Tregs is necessary for modulating Th1 immunity [26]. The question of whether a similar adaptation occurs in SCA remains unanswered. Future research should concentrate on elucidating the mechanisms underlying Treg plasticity and stability in SCA, with particular emphasis on FoxP3 regulation and IL‐2 signaling. Given that Tregs must balance stability and adaptation in chronic inflammatory environments, it is critical to determine whether their suppressive function is maintained or altered in SCA. Furthermore, additional studies should aim to evaluate not only the frequency but also the functional efficacy of Tregs, taking into account the various Treg subsets, and explore their interplay with other immune cell types, such as Th17 cells. The development of targeted immunomodulatory strategies, including IL‐2‐based therapies, may provide novel avenues for restoring immune balance and mitigating inflammation‐associated complications in SCA.

## Funding

This work was supported by Baskent Üniversitesi.

## Ethics Statement

The study was approved by the Başkent University Institutional Review Board for Non‐Interventional Clinical Studies (Approval No: 23/12, Date: 18/01/2023) and conducted in accordance with the Declaration of Helsinki.

## Consent

Written informed consent was obtained from all participants or their legal guardians prior to study enrollment.

## Conflicts of Interest

The authors declare no conflicts of interest.

## Supporting information


**Figure S1:** jcla70227‐sup‐0001‐FigureS1.docx. **Flow cytometric gating strategy for identification of T lymphocyte subsets and regulatory T cells**. Representative plots from a patient with sickle cell anemia (SCA) illustrate the sequential gating strategy used for immunophenotyping, as described in the Methods section. The steps are as follows: (a) Gating of lymphocytes on the CD45/SS plot (35.62% of total events, Gate L). (b) Gating of CD3^+^ T cells within the lymphocyte population (42.16% of lymphocytes, Gate M). (c) Identification of CD4^+^ lymphocytes from the CD3^+^ T‐cell population (51.84% of CD3^+^ T cells, Gate N). (d) Gating of CD4^+^CD25^+^ T cells within the CD4^+^ population (9.63% of CD4^+^ T cells, Gate O). € Gating of FoxP3‐positive cells within the CD4^+^CD25^+^ population (9.67% of CD4^+^CD25^+^ T cells, Gate P). (f) Gating of CD8^+^ T lymphocytes within the CD3^+^ T‐cell population (43.35% of CD3^+^ T cells, Gate Q). The percentages indicated in each plot represent the proportion of the parent gate.

**Table S1:** Hematological parameters of the study participants.
**Table S2:** Flow cytometric analysis of T cell subsets in sickle cell anemia patients.
**Table S3:** Hematological parameters in sickle cell anemia patients.

## Data Availability

The data that support the findings of this study are available from the corresponding author upon reasonable request.
