# Supplementary material for: Immune Imbalance in Sickle Cell Anemia: Flow Cytometric Insights Into Regulatory T Cells and Neutrophil Dynamics
Source: J Clin Lab Anal. 2026 Apr 14;40(10):e70227. doi: 10.1002/jcla.70227 (PMC13240530; doi:10.1002/jcla.70227)
Supplement: Supplementary file 1 — Figure S1: jcla70227‐sup‐0001‐FigureS1.docx. Flow cytometric gating strategy for identification of T lymphocyte subsets and regulatory T cells. Representative plots from a patient with sickle cell anemia (SCA) illustrate the sequential gating strategy used for immunophenotyping, as described in the Methods section. The steps are as follows: (a) Gating of lymphocytes on the CD45/SS plot (35.62% of total events, Gate L). (b) Gating of CD3+ T cells within the lymphocyte population (42.16% of lymphocytes, Gate M). (c) Identification of CD4+ lymphocytes from the CD3+ T‐cell population (51.84% of CD3+ T cells, Gate N). (d) Gating of CD4+CD25+ T cells within the CD4+ population (9.63% of CD4+ T cells, Gate O). € Gating of FoxP3‐positive cells within the CD4+CD25+ population (9.67% of CD4+CD25+ T cells, Gate P). (f) Gating of CD8+ T lymphocytes within the CD3+ T‐cell population (43.35% of CD3+ T cells, Gate Q). The percentages indicated in each plot represent the proportion of the parent gate. [file JCLA-40-e70227-s002.docx]

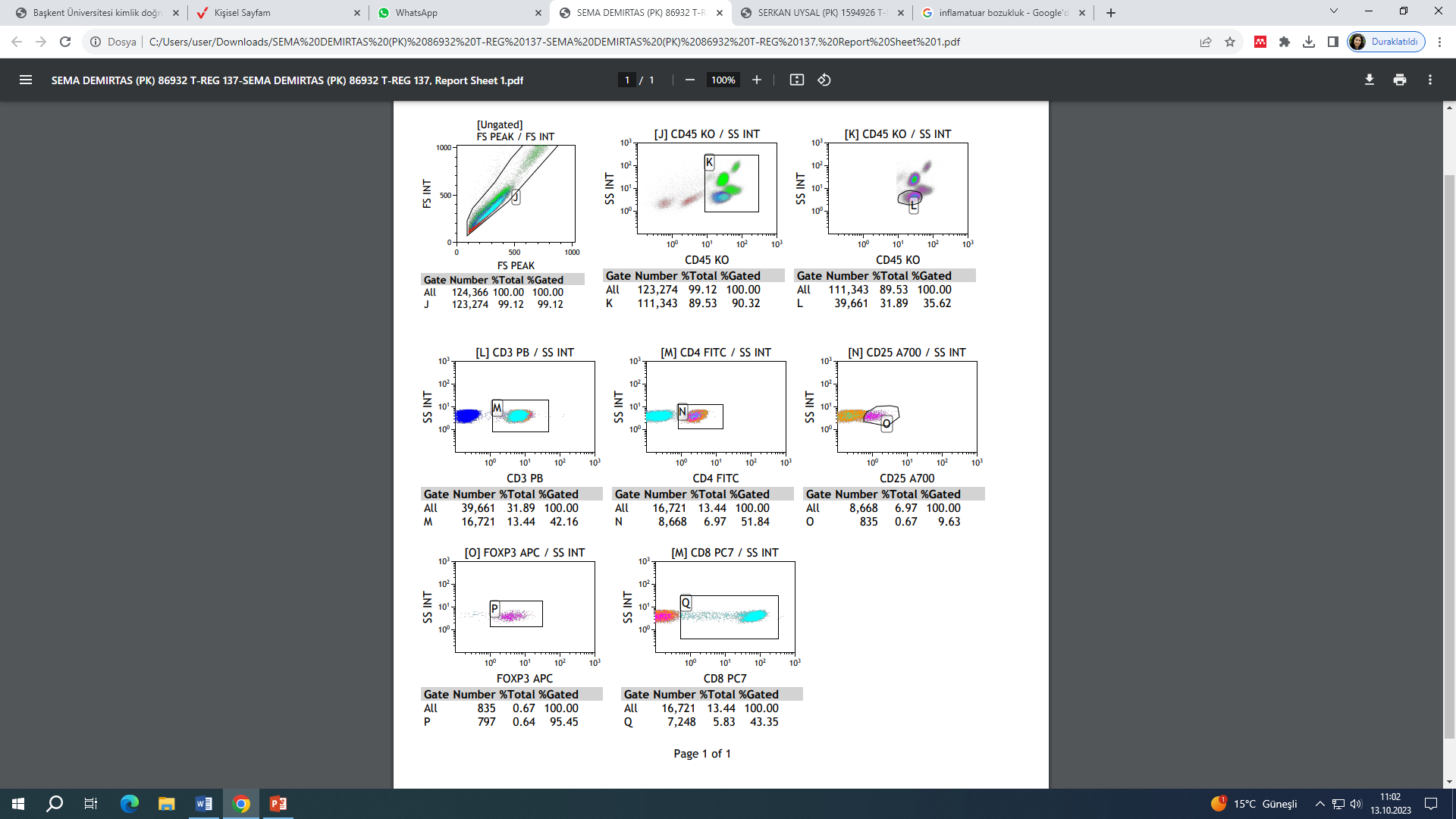


f

e

d

c

b

a

**Figure S1.** **Flow cytometric gating strategy for identification of T lymphocyte subsets and regulatory T cells.** *Representative plots from a patient with sickle cell anemia (SCA) illustrate the sequential gating strategy used for immunophenotyping, as described in the Methods section. The steps are as follows: a) Gating of lymphocytes on the CD45/SS plot (35.62% of total events, Gate L). b) Gating of CD3⁺ T cells within the lymphocyte population (42.16% of lymphocytes, Gate M). c) Identification of CD4⁺ lymphocytes from the CD3⁺ T-cell population (51.84% of CD3⁺ T cells, Gate N). d) Gating of CD4⁺CD25⁺ T cells within the CD4⁺ population (9.63% of CD4⁺ T cells, Gate O). e) Gating of FoxP3-positive cells within the CD4⁺CD25⁺ population (9.67% of CD4⁺CD25⁺ T cells, Gate P). f) Gating of CD8⁺ T lymphocytes within the CD3⁺ T-cell population (43.35% of CD3⁺ T cells, Gate Q).* *The percentages indicated in each plot represent the proportion of the parent gate.*
