# Supplementary material for: Immune Imbalance in Sickle Cell Anemia: Flow Cytometric Insights Into Regulatory T Cells and Neutrophil Dynamics
Source: J Clin Lab Anal. 2026 Apr 14;40(10):e70227. doi: 10.1002/jcla.70227 (PMC13240530; doi:10.1002/jcla.70227)
Supplement: Supplementary file 2 — Table S1: Hematological parameters of the study participants. Table S2: Flow cytometric analysis of T cell subsets in sickle cell anemia patients. Table S3: Hematological parameters in sickle cell anemia patients. [file JCLA-40-e70227-s001.docx]

**Table S1**. **Hematological parameters of the study participants.**

|  | **HG (n=49)** | | | **SCA_PC (n=17)** | | | **SCA_PC - HG** | | **SCA_SS (n=27)** | | | **SCA_SS –HG** | | **SCA (n=44)** | | **SCA – HG** | | |
| --- | --- | --- | --- | --- | --- | --- | --- | --- | --- | --- | --- | --- | --- | --- | --- | --- | --- | --- |
| **Parameters** | Mean±SD | Median (IQR) | Mean±SD | | Median (IQR) | **Test statistics** | | **p** | Mean±SD | Median (IQR) | **Test statistics** | | **p** | Mean±SD | Median (IQR) | | **Test statistics** | **p** |
| WBC (White blood cells) | 7.25±1.76 | 7 (2.46) | 17.75±10.20 | | 15.50 (8) | -4.421^Z^ | | **<0.001***** | 11.69±5.42 | 10.80 (5.98) | -4.385^Z^ | | **<0.001***** | 14.03±8.08 | 13.40 (8.61) | | -5.429^Z^ | **<0.001***** |
| Neutrophil% | 57.42±7.86 | 58.70 (13.8) | 35.29±17.48 | | 35 (31) | -5.048^t^ | | **<0.001***** | 45.76±15.52 | 44.6 (24.9) | -4.360^t^ | | **<0.001***** | 41.71±16.91 | 42.30 (24.95) | | -5.641^t^ | **<0.001***** |
| Lymphocyte% | 31.66±7.39 | 32.10 (12.10) | 50.73±18.80 | | 57.30 (34.1) | 4.075^t^ | | **0.001***** | 43.21±15.05 | 44.4 (29.4) | 4.493^t^ | | **<0.001***** | 46.11±16.80 | 45.70 (29.50) | | 5.465^t^ | **<0.001***** |
| Monocyte% | 7.27±2.14 | 7.13 (3.12) | 9.00±3.40 | | 8.61 (4.21) | 2.438^t^ | | **0.018*** | 7.38±2.96 | 6.97 (4.67) | 0.172^t^ | | 0.864 | 8.00±3.20 | 8.09 (4.71) | | 1.277^t^ | 0.206 |
| HGB (Hemoglobin) | 13.82±1.59 | 13.4 (2.45) | 8.74±2.13 | | 8.50 (2.11) | -5.479^Z^ | | **<0.001***** | 8.93±2.39 | 8.46 (3.91) | -10.680^t^ | | **<0.001***** | 8.86±2.27 | 8.48 (3.17) | | -7.519^Z^ | **<0.001***** |
| HCT (Hematocrit) | 40.66±7.78 | 42 (7.05) | 26.94±6.69 | | 25.20 (7.1) | -4.927^Z^ | | **<0.001***** | 28.55±7.79 | 27 (10.2) | -5.612^Z^ | | **<0.001***** | 27.93±7.35 | 26.55 (9.60) | | -6.564^Z^ | **<0.001***** |
| PLT (Platelets) | 256.94±64.43 | 253 (89) | 374.12±170.81 | | 345 (250) | 2.761^t^ | | **0.013*** | 384.30±198.23 | 391 (277) | 4.137^t^ | | **<0.001***** | 380.37±186.12 | 359.50 (267.50) | | 4.180^t^ | **<0.001***** |

*The data are presented as mean ± standard deviation and median (interquartile range-IQR). Group comparisons were performed using either the Independent Samples t-test (marked 't') or the Mann-Whitney U test (marked 'Z'), as appropriate. HG = healthy controls; SCA_PC = SCA patients in painful crisis; SCA_SS = SCA patients in steady state; SCA = all SCA patients combined; WBC = white blood cells; HGB = haemoglobin; HCT = haematocrit; PLT = platelets; *p<0.05, **p<0.01, ***p<0.001*

**Table S2. Flow cytometric analysis of T cell subsets in sickle cell anemia patients.**

| **SCA (n=44)** |  | Lymphocyte % | CD3+ % | CD3+ CD4+ CD25+ % | CD3+ CD4+ CD25+ count | CD3+ CD4+ CD25+ FoxP3+ % | CD3+ CD4+ CD25+ FoxP3+ count | CD3+ CD4+ (T helper) % | CD3+ CD8+ (Cytotoxic T) % | CD4/CD8 ratio | Treg (CD4+ CD25+ FoxP3+) % in total cells | CD3+ CD4+ CD25+ % in total cells |
| --- | --- | --- | --- | --- | --- | --- | --- | --- | --- | --- | --- | --- |
|  |  | Mean±SD | Mean±SD | Mean±SD | Mean±SD | Mean±SD | Mean±SD | Mean±SD | Mean±SD | Mean±SD | Mean±SD | Mean±SD |
|  |  | Median (IQR) | Median (IQR) | Median (IQR) | Median (IQR) | Median (IQR) | Median (IQR) | Median (IQR) | Median (IQR) | Median (IQR) | Median (IQR) | Median (IQR) |
| **Cerebrovascular Event** | No (n=40) | 38.67±11.69  40.24 (16.29) | 62.78±11.19  62.33 (17.50) | 2.31±1.45  2.13 (1.87) | 532.95±334.85  484 (449.50) | 4.46±1.83  4.66 (2.90) | 1036.55±470.04  998 (814) | 37.96±8.23  37.51 (10.25) | 20.53±7.39  19.37 (10.12) | 2.09±0.85  1.97 (1.45) | 1.04±0.52  1.01 (0.70) | 0.53±0.32  0.49 (0.46) |
|  | Yes (n=4) | 33.17±4.09  33.36 (7.68) | 52.53±11.79  50.16 (22.14) | 2.94±1.93  2.16 (3.16) | 520.50±338.78  437 (625) | 5.65±1.10  5.53 (2.03) | 980±227.91  999.50 (413.50) | 33.81±8.53  36.83 (15.03) | 15.27±8.63  15.37 (16.62) | 3.37±3.16  2.11 (5.37) | 0.96±0.19  0.98 (0.37) | 0.50±0.29  0.43 (0.54) |
|  | *p* | 0.242^U^ | 0.079^U^ | 0.459^U^ | 0.984^U^ | 0.133^U^ | 0.845^U^ | 0.546^U^ | 0.293^U^ | 0.800^U^ | 0.938^U^ | 1.000^U^ |
| **Avascular Necrosis** | No (n=20) | 36.31±12.88  37.74 (17.67) | 63.20±11.48  63.22 (17.34) | 2.28±1.12  2.13 (1.52) | 521±345.44  469.50 (519.25) | 4.70±1.73  4.86 (2.35) | 1033.95±542.50  947 (873) | 37.22±7.97  38.60 (8.72) | 21.96±8.30  20.85 (14.21) | 1.97±0.91  1.59 (1.48) | 1.07±0.60  1.09 (0.92) | 0.53±0.35  0.52 (0.53) |
|  | Yes (n=24) | 39.91±9.63  40.60 (13.64) | 60.69±11.60  57.83 (19.14) | 2.44±1.77  1.93 (2.50) | 541.83±325.12  484 (444.25) | 4.44±1.89  4.64 (2.73) | 1029.50±363.87  999.50 (556.25) | 37.95±8.65  36.59 (10.57) | 18.35±6.48  17.63 (7.38) | 2.42±1.41  2.14 (1.44) | 0.99±0.40  0.96 (0.51) | 0.52±0.30  0.45 (0.46) |
|  | *p* | 0.286^t^ | 0.466^t^ | 0.715^t^ | 0.834^t^ | 0.632^t^ | 0.974^t^ | 0.768^t^ | 0.105^t^ | 0.226^U^ | 0.606^t^ | 0.859^t^ |
| **Retinopathy** | No (n=40) | 38.08±11.71  39.68 (17.11) | 61.16±11.47  60.84 (17.81) | 2.34±1.52  2.10 (1.79) | 514.48±334  460 (464.75) | 4.60±1.84  4.67 (3.11) | 1021.07±467.75  925 (814) | 37.04±8.22  36.68 (8.83) | 19.77±7.61  19.20 (9.29) | 2.22±1.24  1.97 (1.45) | 1.03±0.52  0.95 (0.70) | 0.51±0.32  0.45 (0.46) |
|  | Yes (n=3) | 36.41±3.94  35.63 (-) | 69.17±11.87  71.52 (-) | 2.63±1.33  3.00 (-) | 680±322.31  729.00 (-) | 4.53±1.40  5.03 (-) | 1182.33±302.27  1220 (-) | 40.60±4.71  38.64 (-) | 25.40±6.77  29.28 (-) | 1.67±0.41  1.57 (-) | 1.10±0.23  1.18 (-) | 0.63±0.27  0.71 (-) |
|  | *p* | 0.682^U^ | 0.219^U^ | 0.665^U^ | 0.363^U^ | 0.964^U^ | 0.467^U^ | 0.413^U^ | 0.187^U^ | 0.400^U^ | 0.509^U^ | 0.387^U^ |
| **Hepatopathy** | No (n=33) | 37.71±10.51  37.23 (15.96) | 63.55±10.47  64.13 (17.33) | 2.33±1.56  2.10 (1.76) | 531.46±335.05  487 (485) | 4.40±1.86  4.66 (3.27) | 1006.51±444.49  874 (713) | 38.07±7.34  38.64 (9.76) | 21.06±7.33  19.88 (10.57) | 2.15±1.27  1.87 (1.46) | 1.01±0.49  0.90 (0.70) | 0.53±0.33  0.42 (0.47) |
|  | Yes (n=10) | 41.97±13.04  43.13 (13.04) | 55.16±13.22  55.05 (12.75) | 2.28±1.20  2.01 (2.22) | 516.70±348.88  431 (545.50) | 4.70±1.01  4.67 (1.71) | 1073.60±500.87  999.50 (746) | 34.85±10.68  34.87 (9.49) | 16.71±6.51  14.94 (10.03) | 2.36±1.05  2.01 (1.76) | 1.13±0.55  1.10 (0.56) | 0.52±0.30  0.50 (0.51) |
|  | *p* | 0.172^U^ | **0.041^U*^** | 0.785^U^ | 0.913^U^ | 0.946^U^ | 0.604^U^ | 0.120^U^ | 0.096^U^ | 0.453^U^ | 0.397^U^ | 0.978^U^ |
| **Nephropathy** | No (n=28) | 38.76±11.14  39.94 (14.84) | 62.72±11.71  61.39 (15.94) | 2.10±1.16  2.10 (1.48) | 490.37±309.77  371.50 (485) | 4.38±1.70  4.66 (2.87) | 997.17±455.28  867 (819) | 37.15±8.11  37.28 (8.49) | 21.27±8.04  20.52 (12.31) | 2.11±1.36  1.76 (1.35) | 1.04±0.55  0.90 (0.75) | 0.50±0.30  0.40 (0.46) |
|  | Yes (n=15) | 38.46±11.05  38.71 (16.31) | 59.61±11.26  56.53 (19.43) | 2.76±1.93  2.22 (2.09) | 603.80±378.20  525 (482) | 4.65±1.74  4.90 (3.18) | 1069.93±458.74  1023 (503) | 37.77±8.56  39.05 (13.69) | 17.75±6.33  16.82 (10.59) | 2.37±0.89  2.31 (1.53) | 1.02±0.42  1.01 (0.54) | 0.58±0.36  0.51 (0.48) |
|  | *p* | 0.866^U^ | 0.386^U^ | 0.360^U^ | 0.263^U^ | 0.588^U^ | 0.523^U^ | 0.588^U^ | 0.155^U^ | 0.135^U^ | 0.782^U^ | 0.434^U^ |
| **Priapism** | No (n=39) | 38.85±11.17  39.89 (14.81) | 62.62±11.52  63.20 (17.14) | 2.31±1.52  2.10 (1.78) | 528.56±336.26  481 (423.50) | 4.44±1.74  4.66 (2.95) | 1038.66±469.15  1020 (798) | 37.77±8.31  37.65 (9.32) | 20.73±7.36  19.46 (11.16) | 2.05±0.81  1.90 (1.43) | 1.05±0.52  1.01 (0.72) | 0.53±0.32  0.48 (0.41) |
|  | Yes (n=4) | 36.65±9.99  37.69 (18.42) | 52.03±6.62  50.06 (12.04) | 2.47±0.97  2.37 (1.74) | 524.25±360.96  488 (653.75) | 4.73±1.36  4.82 (2.56) | 844.75±151.98  851 (288.25) | 33.13±5.61  33.62 (10.81) | 13.59±8.30  12.82 (15.98) | 3.70±3.10  2.73 (5.59) | 0.85±0.15  0.86 (0.29) | 0.52±0.34  0.50 (0.61) |
|  | *p* | 0.690^U^ | **0.046^U*^** | 0.460^U^ | 0.968^U^ | 0.550^U^ | 0.485^U^ | 0.209^U^ | 0.111^U^ | 0.359^U^ | 0.550^U^ | 0.921^U^ |
| **Pulmonary Complication** | No (n=36) | 39.13±11.66  40.24 (16.89) | 62.90±11.26  63.23 (17.04) | 2.21±1.30  2.10 (1.64) | 523.42±332.15  463 (487) | 4.31±1.72  4.66 (3) | 1021.08±487.36  925 (833.50) | 37.52±8.69  38.11 (9.81) | 20.90±7.24  19.29 (10.64) | 2.01±0.82  1.89 (1.45) | 1.04±0.54  0.95 (0.80) | 0.52±0.32  0.47 (0.50) |
|  | Yes (n=7) | 36.09±6.16  35.63 (9.99) | 55.07±11.48  51.32 (8.60) | 2.94±2.22  2.22 (2.27) | 554±371.13  481 (465) | 5.32±1.43  5.53 (1.66) | 1023.29±192.33  1023 (370) | 36.44±4.73  34.40 (5.45) | 15.71±8.78  11.87 (13.36) | 3.20±2.31  2.72 (2.28) | 1.00±0.17  1.02 (0.37) | 0.54±0.35  0.48 (0.49) |
|  | *p* | 0.416^U^ | **0.052^U*^** | 0.521^U^ | 0.913^U^ | 0.194^U^ | 0.754^U^ | 0.650^U^ | 0.125^U^ | 0.121^U^ | 0.695^U^ | 0.963^U^ |
| **Routine Exchange transfusion** | No (n=35) | 38.21±11.08  39.47 (16.97) | 63.28±10.25  63.25 (17.14) | 2.36±1.54  2.16 (1.73) | 543.59±326.26  500 (426.50) | 4.48±1.91  4.66 (3.18) | 1056.24±463.37  1023 (802) | 38.08±7.46  38.56 (9.58) | 20.89±7.42  19.46 (10.42) | 2.07±0.83  2.03 (1.51) | 1.04±0.49  1.02 (0.70) | 0.53±0.44  0.49 (0.44) |
|  | Yes (n=9) | 38.12±12.93  39.89 (15.76) | 56.19±14.96  56.30 (19.66) | 2.36±1.28  2.10 (2.49) | 483.67±368.11  331 (632.50) | 4.90±1.30  5.61 (2.03) | 930.44±415.61  828 (605) | 35.63±11.26  35.33 (11.40) | 16.73±7.55  14.95 (11.72) | 2.76±2.16  1.90 (2.03) | 1.01±0.55  0.89 (0.57) | 0.49±0.32  0.42 (0.57) |
|  | *p* | 0.923^U^ | 0.124^U^ | 0.740^U^ | 0.430^U^ | 0.533^U^ | 0.346^U^ | 0.245^U^ | 0.170^U^ | 0.658^U^ | 0.688^U^ | 0.648^U^ |
| **Alloimmuni-zation** | No (n=35) | 38.55±11.53  39.98 (17.42) | 62.04±11.94  61.45 (17.81) | 2.35±1.58  2.10 (1.98) | 526.27±340.11  439 (463) | 4.62±1.87  4.66 (3.01) | 1044.54±469.59  863 (835) | 37.51±8.68  37.19 (10.91) | 20.20±7.66  19.28 (9.11) | 2.20±1.27  2.03 (1.39) | 1.05±0.53  0.90 (0.70) | 0.52±0.33  0.42 (0.48) |
|  | Yes (n=9) | 36.70±10.89  36.84 (14.66) | 61.30±10.01  56.53 (18.44) | 2.40±1.02  2.22 (1.65) | 554.89±310.63  520 (461) | 4.34±1.57  4.76 (3.03) | 978.56±396.23  1023 (451.50) | 37.99±6.58  38.64 (6.50) | 19.57±7.49  16.82 (14.89) | 2.22±0.92  1.90 (1.70) | 0.96±0.38  1.01 (0.47) | 0.54±0.28  0.52 (0.45) |
|  | *p* | 0.793^U^ | 0.857^U^ | 0.589^U^ | 0.608^U^ | 0.868^U^ | 0.890^U^ | 0.890^U^ | 0.771^U^ | 0.608^U^ | 0.912^U^ | 0.608^U^ |
| **Hydroxyurea** | No (n=12) | 36.15±12.10  35.38 (15.39) | 61.24±14.02  60.91 (21.01) | 2.52±1.33  2.35 (1.92) | 535.93±290.88  536.50 (433.25) | 5.14±1.65  5.65 (2.21) | 1080.79±482.35  921 (888) | 34.69±9.50  35.63 (12.51) | 21.81±9.78  18.57 (15.41) | 1.90±0.96  1.41 (1.81) | 1.11±0.58  0.91 (0.92) | 0.54±0.31  0.50 (0.45) |
|  | Yes (n=31) | 39.10±11.21  40.69 (16.29) | 62.36±10.55  61.45 (17.12) | 2.29±1.58  1.79 (1.89) | 531.68±357.74  383 (514) | 4.27±1.85  4.66 (2.75) | 1004.06±451.52  976 (685) | 39.02±7.52  38.64 (9.16) | 19.21±6.44  19.12 (8.64) | 2.37±1.30  2.15 (1.20) | 0.99±0.47  1.01 (0.54) | 0.52±0.33  0.42 (0.53) |
|  | *p* | 0.292^U^ | 0.961^U^ | 0.418^U^ | 0.797^U^ | 0.135^U^ | 0.864^U^ | 0.135^U^ | 0.750^U^ | 0.211^U^ | 0.844^U^ | 0.797^U^ |
| **Painful crisis** | No (n=9) | 34.47±15.84  32.92 (26.85) | 63.81±11.94  70.31 (19.06) | 2.46±1.30  2.22 (1.34) | 529.00±387.99  481 (730) | 5.22±1.13  5.53 (1.43) | 1099.33±500.91  1220 (762.50) | 39.20±4.16  39.05 (3.69) | 22.33±10.44  22.71 (14.29) | 2.50±2.19  1.51 (1.59) | 1.18±0.66  1.19 (0.71) | 0.56±0.41  0.49 (0.88) |
|  | 1-2 (n=27) | 41.16±10.07  43.26 (11.37) | 63.43±10.56  63.25 (17.33) | 2.05±1.16  1.62 (1.89) | 511.70±291.48  487 (367) | 4.23±2.65  4.65 (2.65) | 1064.59±434.86  976 (696) | 38.18±8.23  35.98 (11.32) | 20.11±7.37  19.88 (8.95) | 2.18±0.91  2.15 (1.53) | 1.05±0.46  1.00 (0.70) | 0.50±2.71  0.48 (0.23) |
|  | ≥3 (n=10) | 33.53±7.72  33.40 (14.16) | 56.01±12.70  56.81 (18.11) | 3.09±2.15  2.55 (2.82) | 588.90±406.24  563.50 (774.25) | 4.86±2.25  5.28 (4.69) | 881.70±472.19  676 (859.50) | 34.62±10.75  34.74 (15.74) | 17.94±4.57  16.94 (5.98) | 2.01±0.69  1.87 (1.39) | 0.86±0.42  0.74 (0.76) | 0.56±0.36  0.56 (0.65) |
|  | *p* | 0.071^KW^ | 0.253^KW^ | 0.309^KW^ | 0.959^KW^ | 0.233^KW^ | 0.351^KW^ | 0.345^KW^ | 0.505^KW^ | 0.760^KW^ | 0.369^KW^ | 0.977^KW^ |

*The data are presented as mean ± standard deviation and median (interquartile range). Group comparisons (Yes vs. No for each complication) were performed using the Independent Samples t-test (marked as 't'), the Mann-Whitney U test (marked as 'U'), or the Kruskal-Wallis test (marked as 'KW' for VOC frequency subgroups), as appropriate. *p<0.05 indicates statistical significance.*

**Table S3.** **Hematological parameters in sickle cell anemia patients.**

| **SCA (n=44)** |  | WBC | Neutrophil % | Lymphocyte % | Monocyte % | HGB | HCT | PLT |
| --- | --- | --- | --- | --- | --- | --- | --- | --- |
|  |  | Mean±SD | Mean±SD | Mean±SD | Mean±SD | Mean±SD | Mean±SD | Mean±SD |
|  |  | Median (IQR) | Median (IQR) | Median (IQR) | Median (IQR) | Median (IQR) | Median (IQR) | Median (IQR) |
| **Cerebrovascular Event** | No (n=40) | 13.86±8.37  12.75 (9.13) | 41.19±17.10  42.20 (24.80) | 46.64±16.84  45.70 (29.33) | 7.90±3.16  7.99 (4.81) | 9.06±2.46  8.68 (3.44) | 28.72±8.02  26.95 (10.13) | 383.22±190.13  382.50 (280.25) |
|  | Yes (n=4) | 11.36±4.75  12.50 (8.83) | 44.73±11.16  44.45 (21.08) | 41.33±13.87  39.55 (26.17) | 9.69±2.96  9.78 (5.60) | 9.05±2.39  8.48 (4.44) | 27.08±6.15  25.55 (11.33) | 296.75±32.28  292.50 (59.75) |
|  | *P* | 0.755^U^ | 0.626^U^ | 0.508^U^ | 0.258^U^ | 0.876^U^ | 0.697^U^ | 0.495^U^ |
| **Avascular Necrosis** | No (n=20) | 13.53±9.55  10.75 (9.09) | 45.23±19.68  48 (26.88) | 43.60±19.27  38.60 (29.50) | 7.09±2.81  7.27 (5.03) | 9.47±2.71  8.24 (4.50) | 30.78±9.00  29.40 (16.30) | 344.21±183.44  292.50 (297.25) |
|  | Yes (n=24) | 13.75±6.75  13.45 (8.37) | 38.07±12.67  38.10 (17.78) | 48.53±13.56  48.55 (21.35) | 8.95±3.24  8.57 (4.61) | 8.69±2.12  8.90 (2.37) | 26.56±6.09  26.55 (6.97) | 404.56±182.27  382.50 (237.88) |
|  | *P* | 0.409^U^ | 0.155^t^ | 0.318^t^ | **0.044^t*^** | 0.605^U^ | 0.073^t^ | 0.270^t^ |
| **Retinopathy** | No (n=40) | 13.53±8.35  11.85 (8.53) | 41.45±16.97  41.25 (25.45) | 46.63±16.63  47.10 (29.10) | 7.78±3.13  7.68 (4.62) | 9.09±2.42  8.48 (3.47) | 28.67±7.67  26.95 (10.25) | 372.27±186.58  334 (254.50) |
|  | Yes (n=3) | 17.17±4.02  16.70 (-) | 41.07±17.31  50.30 (-) | 42.57±20.71  33.20 (-) | 11.23±1.76  11.40 (-) | 8.64±3.39  9.22 (-) | 28.03±13.04  29.80 (-) | 402.67±198.23  496 (-) |
|  | *p* | 0.187^U^ | 1.000^U^ | 0.750^U^ | **0.050^U*^** | 1.000 | 0.909^U^ | 0.649^U^ |
| **Hepatopathy** | No (n=33) | 11.63±6.22  10.80 (6.76) | 45.04±12.98  44.60 (21.70) | 42.91±14.26  41.70 (26.50) | 7.97±2.99  8.07 (4.47) | 9.45±2.58  8.86 (3.62) | 30.15±8.09  28.70 (12) | 391.68±190.98  391 (246) |
|  | Yes (n=10) | 20.16±10.79  16.85 (14.13) | 27.46±2.12  24.53 (20.98) | 59.13±18.59  63.95 (15.32) | 8.37±3.95  8.24 (6.64) | 7.87±1.36  8.39 (2.55) | 23.56±4.33  23.91 (7.75) | 322.85±161.46  264 (249.25) |
|  | *p* | **0.008^U**^** | **0.001^U***^** | **0.004^U**^** | 0.743 | **0.101** | **0.015^U*^** | 0.240^U^ |
| **Nephropathy** | No (n=28) | 13.33±8.68  11.85 (9.37) | 41.31±17.40  42.20 (26.43) | 46.12±17.03  45.70 (29.22) | 8.26±3.20  8.14 (4.54) | 9.50±2.38  9.12 (3.67) | 30.59±7.59  29.25 (10.15) | 363.12±197.57  334 (301.25) |
|  | Yes (n=15) | 13.91±7.28  13.40 (6.56) | 40.78±15.50  37.40 (22.70) | 47.31±16.14  47.50 (28.40) | 7.65±3.23  6.97 (4.97) | 8.29±2.43  8.05 (2.16) | 24.89±7.27  23.40 (6.30) | 402.90±161.21  438 (232) |
|  | *p* | 0.665^U^ | 0.613^U^ | 0.563^U^ | 0.516^U^ | 0.081^U^ | **0.011^U*^** | 0.367^U^ |
| **Priapism** | No (n=39) | 13.67±8.24  12.10 (8.38) | 41.60±16.57  41.60 (23.10) | 46.37±16.25  46.70 (27.25) | 7.78±3.12  7.90 (4.72) | 9.04±2.51  8.50 (3.79) | 28.62±8.06  27 (10.75) | 369.58±184.67  328 (276) |
|  | Yes (n=4) | 12.05±8.20  10.48 (15.30) | 36.42±18.87  37.38 (34.44) | 48.03±22.27  45.32 (41.18) | 10.92±2.70  11.51 (5.01) | 9.69±1.63  9.23 (2.95) | 29.33±6.71  26.95 (11.81) | 446.13±205.02  378 (362.13) |
|  | *p* | 0.720^U^ | 0.577^U^ | 0.842^U^ | 0.061^U^ | 0.338^U^ | 0.795^U^ | 0.461^U^ |
| **Pulmonary Complication** | No (n=36) | 13.10±7.78  11.35 (8.43) | 41.91±17.35  42.20 (25.03) | 45.93±16.97  45.70 (29.33) | 7.79±3.12  7.99 (4.84) | 9.35±2.47  8.89 (3.65) | 29.67±7.85  27.45 (11.40) | 380.62±194.43  351 (257.75) |
|  | Yes (n=7) | 15.81±10.39  13.50 (19.34) | 36.95±11.98  38.80 (24.50) | 49.69±14.87  52.30 (30.70) | 9.50±3.38  8.52 (6.73) | 7.72±1.86  8.46 (3.86) | 23.35±6.02  24.42 (12.20) | 353.36±134.49  340 (277) |
|  | *p* | 0.605^U^ | 0.364^U^ | 0.471^U^ | 0.247^U^ | 0.188^U^ | 0.100^U^ | 0.925^U^ |
| **Routine Exchange transfusion** | No (n=35) | 12.21±6.51  11.10 (7.12) | 43.46±14.08  43 (23.15) | 45.22±14.29  44.70 (28.10) | 7.52±2.76  7.46 (4.59) | 9.21±2.63  8.46 (3.90) | 29.17±8.45  26.90 (11.85) | 389.63±184.93  391 (242.50) |
|  | Yes (n=9) | 19.55±11.44  17.20 (14.55) | 33.43±23.86  29.60 (43.85) | 50.11±24.45  57.30 (47.55) | 10.29±3.83  9.95 (6.30) | 8.47±1.19  9.02 (2.03) | 26.13±3.94  27.90 (6.30) | 318.44±174.90  269 (278) |
|  | *p* | **0.033^U*^** | 0.131^U^ | 0.480^U^ | **0.034^U*^** | 0.879^U^ | 0.524^U^ | 0.274^U^ |
| **Alloimmuni-zation** | No (n=35) | 11.64±8.60  11.60 (8.67) | 40.66±16.80  40.90 (26.75) | 47.01±16.84  47.50 (29.56) | 8.22±3.26  8.21 (4.94) | 9.31±2.52  8.87 (3.78) | 29.34±8.05  28.20 (10.95) | 369.03±182.76  340 (266) |
|  | Yes (n=9) | 13.68±6.13  13.70 (7.39) | 44.93±16.29  44.60 (18.55) | 42.74±15.68  41.70 (25.35) | 7.41±2.74  6.78 (4.35) | 8.06±1.77  7.90 (2.25) | 25.44±6.29  24.90 (3.80) | 403.11±194.20  439 (328.50) |
|  | *p* | 0.524^U^ | 0.658^U^ | 0.463^U^ | 0.552^U^ | 0.179^U^ | 0.184^U^ | 0.729^U^ |
| **Hydroxyurea** | No (n=12) | 14.79±9.58  13.55 (9.08) | 44.31±16.09  43.10 (21.55) | 42.87±16.55  43.80 (15.95) | 8.35±3.51  8.06 (4.05) | 8.92±2.49  8.49 (3.86) | 29.28±8.17  27.55 (13.97) | 369.71±170.96  334.50 (293.25) |
|  | Yes (n=31) | 12.75±7.28  11.10 (8.38) | 40.73±17.00  40.90 (24.10) | 47.09±16.58  49.60 (29.70) | 7.96±3.08  8.10 (5.27) | 9.24±2.39  8.86 (3.62) | 28.65±7.61  27 (9.80) | 383.07±192.31  345 (236) |
|  | *p* | 0.469^U^ | 0.462^U^ | 0.391^U^ | 0.980^U^ | 0.524^U^ | 0.932^U^ | 0.902^U^ |
| **Painful crisis** | No (n=7) | 11.20±6.79  10.80 (10) | 50.44±20.63  51.50 (41.70) | 37.06±18.86  29 (36.75) | 8.09±3.52  8.21 (5.98) | 9.43±2.90  8.50 (5.09) | 31.08±9.50  28.70 (15.70) | 279.19±139.07  259 (211) |
|  | 1-2 (n=27) | 12.48±5.86  12.10 (6.27) | 38.01±13.64  38.80 (17.70) | 49.70±13.67  49.40 (22) | 7.91±3.08  7.46 (5.06) | 9.18±2.55  8.87 (3.68) | 28.33±8.08  26.90 (10.80) | 423.31±178.83  438 (233.50) |
|  | ≥3 (n=10) | 18.99±12.12  16.50 (18.61) | 42.83±33.60  47.30 (33.60) | 44.87±19.72  43.15 (39.15) | 8.43±3.31  8.72 (2.88) | 8.41±1.58  8.59 (2.76) | 26.97±5.36  27.50 (8.35) | 334±201.08  259 (281.25) |
|  | *p* | 0.172^KW^ | 0.304^KW^ | 0.130^KW^ | 0.870^KW^ | 0.698^KW^ | 0.556^KW^ | 0.067^KW^ |

*The data are presented as mean ± standard deviation and median (interquartile range). Group comparisons (Yes vs. No for each complication) were performed using the Independent Samples t-test (marked as 't'), the Mann-Whitney U test (marked as 'U'), or the Kruskal-Wallis test (marked as 'KW' for VOC frequency subgroups), as appropriate. WBC, white blood cells; HGB, hemoglobin; HCT, hematocrit; PLT, platelets; IQR, interquartile range. *p<0.05, **p<0.01, ***p<0.001 indicate statistical significance.*
